# Supplementary material for: Efficacy of Heterologous Prime-Boost Vaccination with H3N2 Influenza Viruses in Pre-Immune Individuals: Studies in the Pig Model
Source: Viruses. 2020 Sep 1;12(9):968. doi: 10.3390/v12090968 (PMC7552030; doi:10.3390/v12090968)
Supplement: Supplementary file 1 [file viruses-12-00968-s001.zip › viruses-907446-supplementary.pdf]

Table S1. Tracheal and lung pathology after challenge with A/swine/Missouri/A01840724/2015 (MO15)

Vaccine group	Pig number	Lesion score after challenge		
		Macroscopic pneumonia (%)	Microscopic lesion score	
		Lung	Trachea	Lung
Naïve challenge control PBS-PBS-PBS	2751	2.75	0	5
	2752	0	0	1
	2753	0	0	1
	2754	1.25	0	7
	2755	0.15	2	6.5
	**Mean**	**0.83**	**0.4**	**4.1**
Infection-immune challenge control NC95-PBS-PBS	2785	0	0	1
	2786	1.5	0	0
	2787	0.1	0	1
	2788	0.75	0	6
	2791	5	0	7
	**Mean**	**1.47**	**0**	**3**
Infection-immune vaccinated (Homologous prime-boost) NC95-G08-G08	2774	0	0	1
	2775	0	0	1
	2776	0	0	0
	2777	0	0	0
	2778	0	0	1
	**Mean**	**0**	**0**	**0.6**
Infection-immune vaccinated (Homologous prime-boost) NC95-PA10-PA10	2779	0	0	0
	2780	0.2	0	1
	2781	0	0	1
	2782	0	0	1
	2783	0	0	0
	**Mean**	**0.04**	**0**	**0.6**
Infection-immune vaccinated (Heterologous prime-boost) NC95-G08-PA10	2760	0.75	0	7
	2761	0	0	1
	2762	1.75	0	8
	2763	0	0	1
	2764	0	0	1
	**Mean**	**0.5**	**0**	**3.6**
Infection-immune vaccinated (Heterologous prime-boost) NC95-PA10-G08	2769	0	0	1
	2770	0	0	0
	2771	1.5	0	1
	2772	0.25	0	1
	2773	0	0	1
	**Mean**	**0.35**	**0**	**0.8**

For macroscopic lesion scores, the percentage of the surface affected with pneumonia was estimated visually for each lobe and the total percentage for the entire lung was calculated as an average of percentages of ventral and dorsal surfaces showing tissue consolidation. Microscopic lung lesion scores are composite scores based on the severity of 3 parameters: (1) epithelial damage in intrapulmonary airways (0-3), (2) peribronchiolar lymphocytic cuffing (0-3), (3) neutrophil exudation in bronchioles and alveoli (0-2); Microscopic tracheal lesion scores are based on the severity of epithelial damage (0-2).
